# Crystal structure, Hirshfeld surface analysis and computational study of 2-chloro-*N*-[4-(methyl­sulfan­yl)phen­yl]acetamide

**DOI:** 10.1107/S2056989020002960

**Published:** 2020-03-31

**Authors:** Sitthichok Mongkholkeaw, Apisit Songsasen, Tanwawan Duangthongyou, Kittipong Chainok, Songwut Suramitr, Worawat Wattanathana, Boontana Wannalerse

**Affiliations:** a Department of Chemistry and Center of Excellence for Innovation in Chemistry, Faculty of Science, Kasetsart University, Bangkok, 10900, Thailand; b Materials and Textile Technology, Faculty of Science and Technology, Thammasat University, Pathum Thani, 12120, Thailand; c Department of Chemistry, Faculty of Science, Kasetsart University, Bangkok, 10900, Thailand; d Department of Materials Engineering, Faculty of Engineering, Kasetsart University Bangkok, 10900, Thailand

**Keywords:** 4-methyl­thio­aniline, chloro­acetyl chloride, hydrogen bonds, π–π inter­actions, crystal structure

## Abstract

In the title compound, C_9_H_10_ClNOS, the amide functional group –C(=O)NH– adopts a *trans* conformation with the four atoms nearly coplanar. This conformation promotes the formation of a *C*(4) hydrogen-bonded chain propagating along the [010] direction. The central part of the mol­ecule, including the six-membered ring, the S and N atoms, is fairly planar (r.m.s. deviation of 0.014). The terminal methyl group and the C(=O)CH_2_ group are slightly deviating out-of-plane while the terminal Cl atom is almost in-plane. Hirshfeld surface analysis of the title compound suggests that the most significant contacts in the crystal are H⋯H, H⋯Cl/Cl⋯H, H⋯C/C⋯H, H⋯O/O⋯H and H⋯S/S⋯H. π–π inter­actions between inversion-related mol­ecules also contribute to the crystal packing. DFT calculations have been performed to optimize the structure of the title compound using the CAM-B3LYP functional and the 6–311 G(d,p) basis set. The theoretical absorption spectrum of the title compound was calculated using the TD–DFT method. The analysis of frontier orbitals revealed that the π–π* electronic transition was the major contributor to the absorption peak in the electronic spectrum.

## Abstract 2

In the title compound, the amide functional group –C(=O)NH– adopts a *trans* conformation with the four atoms nearly coplanar. This conformation promotes the formation of a *C*(4) hydrogen-bonded chain propagating along the [010] direction.

## Chemical context   

Methyl­thio­anilines are a class of S- and N- heterocyclic compounds that are widely used in anti­microbial applications (Chatterjee *et al.*, 2012 ▸; Martin *et al.*, 2016 ▸; Das *et al.*, 2017 ▸; Cross *et al.*, 2018 ▸). Metal–methyl­thio­aniline complexes have also been utilized in many applications including as homogeneous catalysts, organic semiconductors, anti­bacterial and anti­fungal drugs (Chen *et al.*, 2019 ▸; Kumar *et al.*, 2017 ▸; Mandal *et al.*, 2018 ▸; Wang *et al.*, 2009 ▸). In this research, we report the synthesis and the solid state structure of 2-chloro-*N*-[4-(methyl­sulfan­yl)phen­yl]acetamide, a methyl­thio­aniline derivative. Hirshfeld surface analysis was used to investigate the inter­actions within the crystal structure and DFT calculations were performed to study the frontier mol­ecular orbitals of the title compound and also its electronic properties.
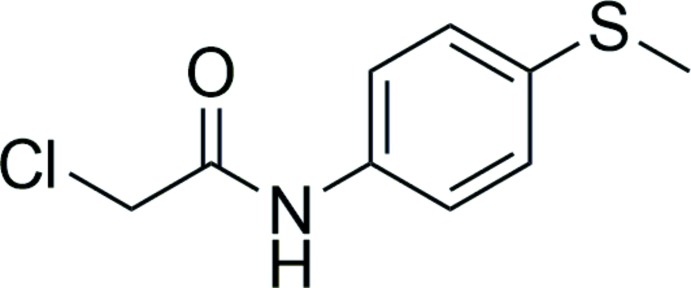


## Structural commentary   

**Figure 1:**
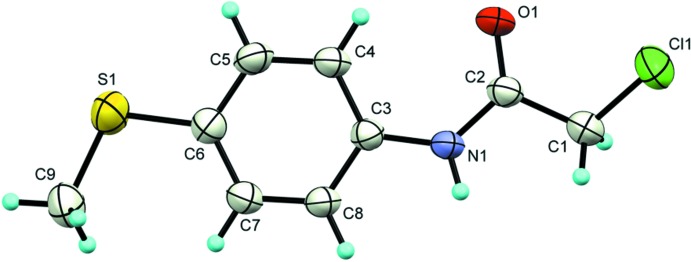
The mol­ecular structure of the title compound, showing the atom-labelling scheme and displacement ellipsoids at the 50% probability level.

The asymmetric unit of the title compound contains one mol­ecule (Fig. 1 ▸). The central part of the mol­ecule, including the six-membered ring, the S and N atoms, is fairly planar (r.m.s. deviation of 0.0142 for the eight fitted non-hydrogen atoms). The terminal methyl group deviates from this plane, atom C9 being displaced by −0.498 (4) Å to the mean plane. On the other side of the benzene ring, the C(=O)CH_2_ group also deviates slightly from the central plane in the opposite direction [deviations of 0.246 (3), 0.324 (3) and 0.489 (4) Å for atoms C2, O1 and C1, respectively] while the terminal Cl atom is almost in-plane [−0.007 (3) Å] as a result of the N1—C2—C1—Cl1 torsion angle of −150.97 (18)°. The amide functional group adopts a *trans* conformation with the four atoms nearly coplanar as shown by the O1—C2—N1—H1 torsion angle of −176.5 (19)°. An intra­molecular C—H⋯O contact is observed (Table 1 ▸).

## Supra­molecular features   

**Figure 2:**
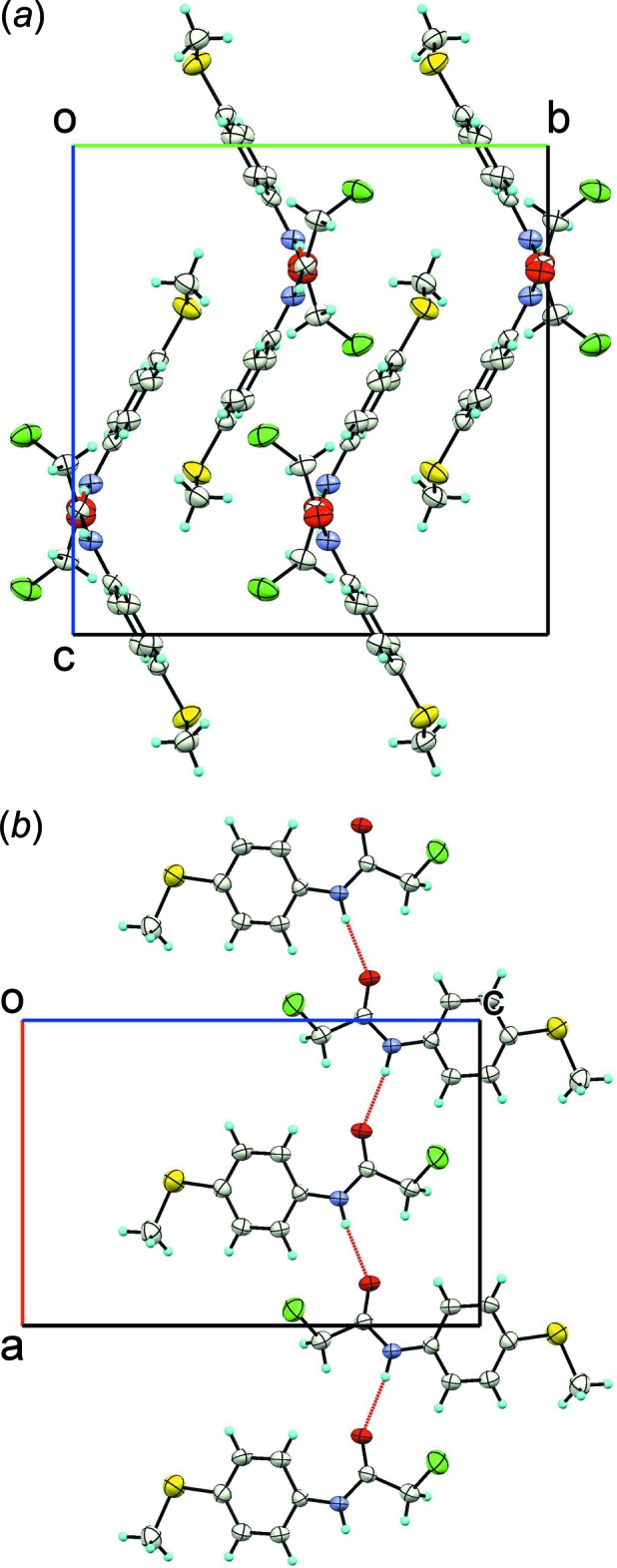
The mol­ecular packing in the title compound: (*a*) view of the unit-cell contents shown in projection down the *a* axis; (*b*) view of the supra­molecular chain perpendicular to the *b* axis originated by the N—H⋯O hydrogen bonding (shown as red dashed lines). Displacement ellipsoids are drawn at the 50% probability level.

The main feature of the crystal packing is the presence of an N—H⋯O hydrogen-bonded chain along the *a-*axis direction (Table 1 ▸) with graph set *C*(4). A view along the *a* axis showing the unit-cell packing is shown in Fig. 2 ▸ *a* while the hydrogen-bonded chain is illustrated in Fig. 2 ▸ *b*. Apart from the hydrogen-bonding inter­actions, π-π stacking is observed between inversion-related mol­ecules. The distance between the ring centroids is 3.8890 (14) Å while the distance between the mean planes is 3.3922 (10) Å (slippage 1.904 Å).

## Hirshfeld analysis   

**Figure 3:**
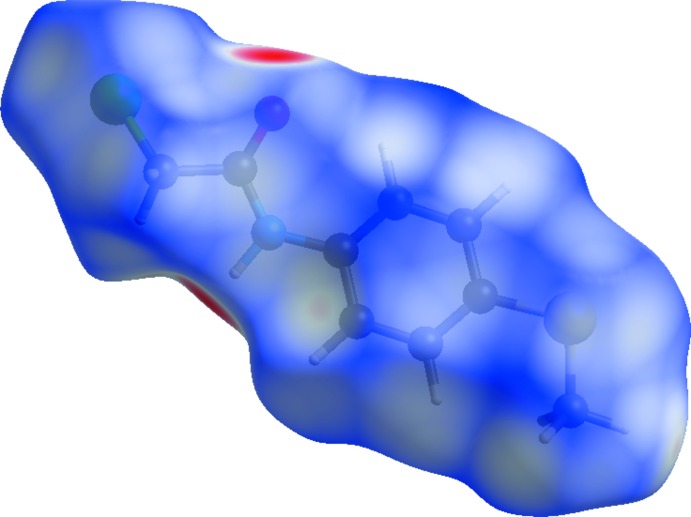
View of the three-dimensional Hirshfeld surface of the title compound plotted over *d*_norm_ in the range −0.5588 to 1.0138 a.u.

**Figure 4:**
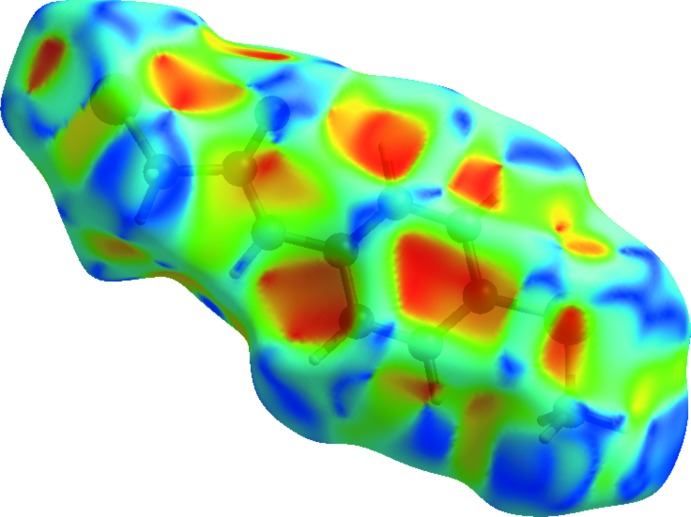
The shape-index Hirshfeld surface of the title compound plotted in the range from −1.0000 to 1.0000 a.u.

**Figure 5:**
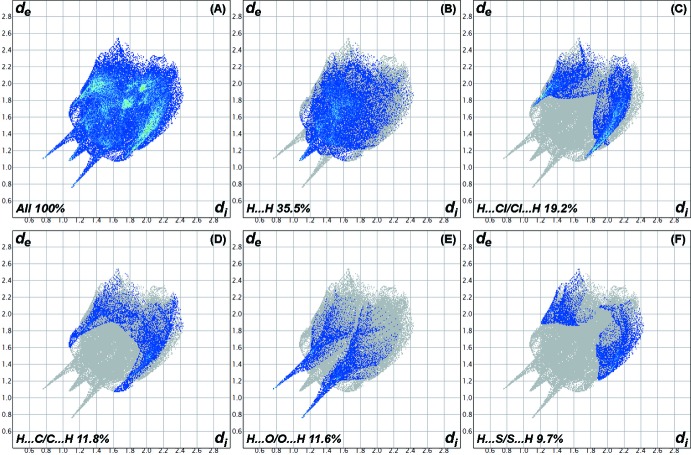
The full two-dimensional fingerprint plot for the title compound, showing (*a*) all inter­actions and those delineated into (*b*) H⋯H, (*c*) H⋯Cl/ Cl⋯H, (*d*) H⋯C/C⋯H, (*e*) H⋯O/O⋯H and (*f*) H⋯S/S⋯H inter­actions. The *d*_i_ and *d*_e_ values are the closest inter­nal and external distances (in Å) from given points on the Hirshfeld surface.

The inter­molecular inter­actions in the crystal of the title compound were investigated by performing a Hirshfeld surface (HS) analysis (Hirshfeld, 1977 ▸; Spackman & Jayatilaka, 2009 ▸) using *Crystal Explorer 17.5* (Turner *et al.*, 2017 ▸). The HS is plotted over the *d*_norm_ range −0.5588 to 1.0138 a.u. (Fig. 3 ▸). The faint red spots on the Hirshfeld surface near atoms O1 and H1 confirm the hydrogen bonding described above. The presence of adjacent orange and blue triangular regions in the shape-index HS (Fig. 4 ▸) confirm that π–π inter­actions also occur in the crystal. Fig. 5 ▸ shows the full two-dimensional fingerprint plot (McKinnon *et al.*, 2007 ▸) and those delineated into H⋯H (35.5%), H⋯Cl/Cl⋯H (19.2%), H⋯C/C⋯H (11.8%), H⋯O/O⋯H (11.6%) and H⋯S/S⋯H (9.7%) contacts. The H⋯H contacts are characterized by a single spike at *d_e_* + *d_i_* ≃ 2.2 Å, while the H⋯O/O⋯H contacts are viewed as a pair of spikes at *d_e_* + *d_i_* ≃ 1.8 Å. Two pairs of beak-shaped tips at *d_e_* + *d_i_* ≃ 2.8 and 3.1 Å represent H⋯Cl/Cl⋯H and H⋯S/S⋯H contacts, respectively. The H⋯C/C⋯H contacts are seen as forcep-like tips at *d_e_* + *d_i_* ≃ 2.7 Å. Other contacts with smaller contributions to the HS have a less significant effect on the crystal packing: C⋯Cl/Cl⋯C (2.9%), C⋯S/S⋯C (2.1%), H⋯N/N⋯H (2.1%) and C⋯C (2.8%).

## Computational Methods   

DFT calculations were carried out to optimize the structure of the title compound using the CAM-B3LYP method and the 6-311G(d,p) basis set in an ethanol solvent within the *Gaussian09* program package (Frisch *et al.*, 2010 ▸). DFT was chosen because it is a good compromise between the computational time and the description of the electronic correlation and has been found to be the best method to obtain accuracy for mol­ecular geometry and electronic transition energies for organic mol­ecules (Perdew *et al.*, 2005 ▸; Niskanen *et al.*, 2014 ▸; Arı *et al.*, 2017 ▸; Miengmern *et al.*, 2019 ▸). Time-dependent density functional theory (TD–DFT) (Jacquemin *et al.*, 2009 ▸) was also used for the calculation of the electronic transitions of the title compound in conjunction with the polarized continuum model (PCM) for computation of the solvent effect (Scalmani *et al.*, 2010 ▸). The theoretical absorption spectrum of the optimized structure of the titled compound in ethanol solvent was obtained using the TD–DFT method. The electronic properties such as *E*_HOMO_, *E*_LUMO_, and the energy gap between HOMO and LUMO of the optimized structure were also determined and the electronic structure of the title compound was visualized in order to understand the hyperconjugative inter­actions and charge delocalization.

## Computational study   

The DFT structure optimization of the compound was performed starting from the X-ray geometry at the CAM-B3LYP/6-311G(d,p) level of theory in an ethanol solvent. The experimental and calculated geometrical parameters such as bond lengths and angles show good agreement although most of the calculated bond lengths are slightly longer than X-ray values (about 0.01 Å) because experimental values are for inter­acting mol­ecules in the crystal lattice, whereas the computational method deals with an isolated mol­ecule in the solvent phase.

**Figure 6:**
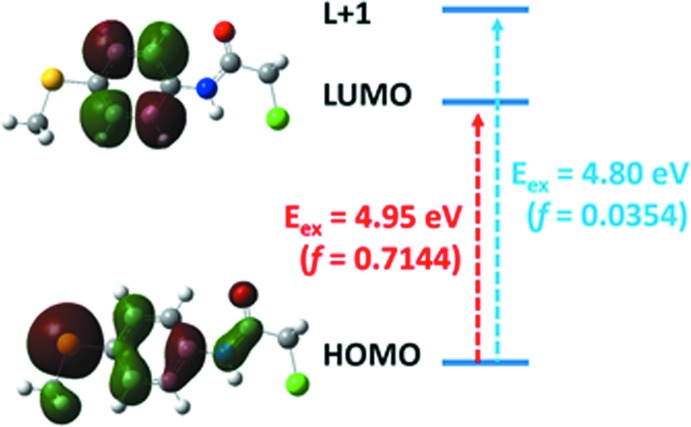
The mol­ecular orbitals (MO) regarding information of the absorption spectrum of the title compound at the *S*_0_→*S*_1_ and *S*_0_→*S*_2_ states calculated by the CAM-B3LYP/6–311 G(d,p) method.

We used the TD-CAM-B3LYP/6-311G(d,p) method to predict the absorption spectrum of the title compound in ethanol, also considering the excited states in the calculation. The maximum absorption wavelength (λ_max_) of the title compound was obtained using this method. As seen in Table 2 ▸, the strong absorption at λ_max_ = 250 nm and the oscillator strength *f* = 0.7144 are due to the *S*_0_→*S*_2_ electronic transition with a wave function of two configurations [(HOMO→LUMO) and (HOMO→*L*+4)]. The transition from HOMO to LUMO is mainly responsible for the formation of the maximum wavelength at 250 nm (Table 3 ▸). Fig. 6 ▸ shows the shape of mol­ecular orbitals participating in the absorption at λ_max_ = 250 nm. The electron density of the HOMO is mainly focused on the –C=C– group in the phenyl ring, the sulfur atom, S–CH_3_, –NH=C and –C=O groups, whereas the LUMO is mainly focused on the =C—C= group in the phenyl ring. Therefore, the electronic transition from HOMO to LUMO mainly corresponds to the π–π* electron. The other excited states of the title compound have a very small intensity that is nearly forbidden by orbital symmetry considerations.

## Database survey   

A search of the Cambridge Structural Database (CSD version 5.41, November 2019 update; Groom *et al.*, 2016 ▸) for the 2-chloro-*N*-phenyl­acetamide core shows that most of the structures were reported by Gowda and Co-workers, for example, the compound 2-chloro-*N*-phenyl­acetamide (I) (RIYWIG; Gowda *et al.*, 2008*a* ▸) reported in the *C*c space group. Other structures with substituent(s) on the benzene ring include 2-chloro-*N*-(2,3-di­chloro­phen­yl)acetamide (II) (GISWEL; Gowda *et al.*, 2008*b* ▸) in the *P*2_1_/*n* space group, 2-chloro-*N*-(3,5-di­chloro­phen­yl)acetamide (III) (GISWIP; Gowda *et al.*, 2008*c* ▸) also in *P*2_1_/*n*, and 2-chloro-*N*-(2,4-di­methyl­phen­yl)acetamide (IV) (YIRJAL; Gowda *et al.*, 2008*d* ▸) in *P* 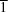. The title compound and compounds (I)–(IV) crystallized in different space groups so it can be concluded that the substituent(s) play a vital role in the crystallization of 2-chloro-*N*-phenyl­acetamide derivatives. It is worth noting that the structures all of the above 2-chloro-*N*-phenyl­acetamide derivatives feature a *C*(4) hydrogen-bond chain involving the primary amide functional group. This feature was also observed in the crystal structures of 2,2-chloro-*N*-phenyl­acetamide derivatives, *viz*. 2,2-di­chloro-*N*-(2,3-di­methyl­phen­yl)acetamide (V) (space group *C*2/*c*; XISROH; Gowda *et al.*, 2008*e* ▸) and 2,2-di­chloro-*N*-(3,5-di­methyl­phen­yl)acetamide (VI) (*P*2_1_/*n*; GISGUL; Gowda *et al.*, 2008*f* ▸) and the crystal structure of the derivative with no substituent on the 2nd position, [*N*-(3-chloro­phen­yl)acetamide] (VII) (*P*2_1_2_1_2_1_; GISPOO; Gowda *et al.*, 2008*g* ▸). This suggests that the substituents on both the benzene ring and at the 2-position did not affect the hydrogen-bonded framework in the *N*-phenyl­acetamide crystal structures. However, the derivatives with an *N,N*-disubstituted acetamide moiety cannot form hydrogen bonds in the same fashion as the *N*-monosubstituted acetamide derivatives because of the lack of a hydrogen-bond donor on the amide nitro­gen atom. The supra­molecular packing of *N*,*N*-disubstituted acetamide derivatives instead features weak inter­molecular C—H⋯O inter­actions (Zhi *et al.*, 2011 ▸).

## Synthesis and crystallization   

The title compound was prepared by combining 4-(methyl­thio)­aniline (5.0 g), chloro­acetyl­chloride (4.30 mL) and tri­ethyl­amine (7.50 mL) in di­chloro­methane (10 mL) at a controlled temperature using an ice bath. After stirring under an N_2_ atmosphere for 24 h, the reaction mixture was poured into water and extracted with 30 mL CH_2_Cl_2_ (3 times). The organic layer was dried with anhydrous Na_2_SO_4_. The mixture product was purified by column chromatography using 9:1 CH_2_Cl_2_/EtOAc as an eluent, affording a light-brown solid, yield 42%. Light-brown crystals were grown by evaporating a solution of the title compound in a mixture of di­chloro­methane and hexane (1:1) at room temperature. ^1^H NMR (CHCl_3_-*d*; 400 MHz): δ 8.23 (1H, *s*, NH), 7.51 (2H, *d*, ArH), 7.29 (2H, *d*, ArH), 4.20 (2H, *s*, CH_2_), 2.50 (3H, *s*, SCH_3_). Analysis calculated: C_9_H_10_ClNOS: C, 50.11; H, 4.67; N, 6.49 Found: C, 50.44; H, 4.69; N, 6.50

## Refinement   

Crystal data, data collection and structure refinement details are summarized in Table 3 ▸. All C-bound H atoms were positioned geometrically and refined using a riding model with *d*(C—H) = 0.95 Å and *U*_iso_(H) = 1.2*U*_eq_(C) for aromatic and *d*(C—H) = 0.98 Å, *U*_iso_(H) = 1.5*U*_eq_(C) for methyl H atoms. The N-bound H atom (H1) was located in a difference-Fourier map and freely refined.

## Supplementary Material

Crystal structure: contains datablock(s) I. DOI: 10.1107/S2056989020002960/zq2250sup1.cif

Structure factors: contains datablock(s) I. DOI: 10.1107/S2056989020002960/zq2250Isup3.hkl

CCDC reference: 1987798

Additional supporting information:  crystallographic information; 3D view; checkCIF report

## Figures and Tables

**Table 1:** Hydrogen-bond geometry (Å, °)

*D*—H⋯*A*	*D*—H	H⋯*A*	*D*⋯*A*	*D*—H⋯*A*
N1—H1⋯O1^i^	0.82 (2)	2.06 (3)	2.875 (2)	174 (3)
C1—H1*B*⋯O1^i^	0.97	2.57	3.319 (3)	135
C4—H4⋯O1	0.93	2.32	2.903 (3)	121

Symmetry code: (i) 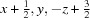.

**Table 2:** The electronic absorption spectrum of the title compound calculated by the TD-CAM-B3LYP/6–311G(d,p) method

Excited states		Excitation energy		Configurations composition
	eV	nm	*f*	
*S*_0_→*S*_1_	4.80	258	0.0354	HOMO→*L*+1 (84%)
*S*_0_→*S*_2_	4.95	250	0.7144	HOMO→LUMO (91%)
*S*_0_→*S*_3_	5.55	224	0.0000	HOMO→*L*+3 (81%)
*S*_0_→*S*_4_	5.63	220	0.0005	*H*−3→LUMO (69%)
*S*_0_→*S*_5_	6.35	195	0.1378	*H*−1→LUMO (59%)

**Table 3:** Experimental details

Crystal data	
Chemical formula	C_9_H_10_ClNOS
*M*_r_	215.69
Crystal system, space group	Orthorhombic, *P* *b* *c* *a*
Temperature (K)	296
*a*, *b*, *c* (Å)	9.6659 (7), 14.0682 (11), 14.4869 (13)
*V* (Å^3^)	1970.0 (3)
*Z*	8
Radiation type	Mo *K*α
μ (mm^−1^)	0.56
Crystal size (mm)	0.12 × 0.10 × 0.08

Data collection	
Diffractometer	Bruker APEXII CCD
Absorption correction	Multi-scan (*SADABS*; Bruker, 2016 ▸)
*T*_min_, *T*_max_	0.686, 0.746
No. of measured, independent and observed [*I* > 2σ(*I*)] reflections	21994, 2438, 1722
*R*_int_	0.085
(sin θ/λ)_max_ (Å^−1^)	0.667

Refinement	
*R*[*F* ^2^ > 2σ(*F* ^2^)], *wR*(*F* ^2^), *S*	0.047, 0.127, 1.02
No. of reflections	2438
No. of parameters	123
H-atom treatment	H atoms treated by a mixture of independent and constrained refinement
Δρ_max_, Δρ_min_ (e Å^−3^)	0.51, −0.41

Computer programs: *APEX2* and *SAINT* (Bruker, 2016 ▸), *SHELXT2014/5* (Sheldrick, 2015*a* ▸), *SHELXL2018/3* (Sheldrick, 2015*b* ▸), *OLEX2* (Dolomanov *et al.*, 2009 ▸) and *Mercury* (Macrae *et al.*, 2020 ▸).

## supplementary crystallographic information

### Crystal data

**Table:**

C_9_H_10_ClNOS	*D*_x_ = 1.454 Mg m^−^^3^
*M**_r_* = 215.69	Mo *K*α radiation, λ = 0.71073 Å
Orthorhombic, *P**b**c**a*	Cell parameters from 3435 reflections
*a* = 9.6659 (7) Å	θ = 2.8–26.2°
*b* = 14.0682 (11) Å	µ = 0.56 mm^−^^1^
*c* = 14.4869 (13) Å	*T* = 296 K
*V* = 1970.0 (3) Å^3^	Block, light brown
*Z* = 8	0.12 × 0.10 × 0.08 mm
*F*(000) = 896	

### Data collection

**Table:**

Bruker APEXII CCD diffractometer	1722 reflections with *I* > 2σ(*I*)
φ and ω scans	*R*_int_ = 0.085
Absorption correction: multi-scan (SADABS; Bruker, 2016)	θ_max_ = 28.3°, θ_min_ = 2.8°
*T*_min_ = 0.686, *T*_max_ = 0.746	*h* = −12→12
21994 measured reflections	*k* = −18→17
2438 independent reflections	*l* = −19→17

### Refinement

**Table:**

Refinement on *F*^2^	Primary atom site location: dual
Least-squares matrix: full	Hydrogen site location: mixed
*R*[*F*^2^ > 2σ(*F*^2^)] = 0.047	H atoms treated by a mixture of independent and constrained refinement
*wR*(*F*^2^) = 0.127	*w* = 1/[σ^2^(*F*_o_^2^) + (0.051*P*)^2^ + 1.0616*P*] where *P* = (*F*_o_^2^ + 2*F*_c_^2^)/3
*S* = 1.02	(Δ/σ)_max_ < 0.001
2438 reflections	Δρ_max_ = 0.51 e Å^−^^3^
123 parameters	Δρ_min_ = −0.41 e Å^−^^3^
0 restraints	

### Special details

**Table:**

Geometry. All esds (except the esd in the dihedral angle between two l.s. planes) are estimated using the full covariance matrix. The cell esds are taken into account individually in the estimation of esds in distances, angles and torsion angles; correlations between esds in cell parameters are only used when they are defined by crystal symmetry. An approximate (isotropic) treatment of cell esds is used for estimating esds involving l.s. planes.

### Fractional atomic coordinates and isotropic or equivalent isotropic displacement parameters (Å^2^)

**Table:**

	*x*	*y*	*z*	*U*_iso_*/*U*_eq_	
Cl1	0.44778 (8)	0.40070 (6)	0.90711 (5)	0.0685 (3)	
S1	0.52668 (8)	0.73979 (6)	0.33195 (5)	0.0612 (2)	
O1	0.36226 (15)	0.51663 (13)	0.74128 (12)	0.0498 (4)	
N1	0.58369 (17)	0.53698 (14)	0.69210 (13)	0.0364 (4)	
H1	0.663 (3)	0.5277 (17)	0.7080 (18)	0.044*	
C3	0.56559 (19)	0.58332 (15)	0.60636 (15)	0.0333 (4)	
C7	0.6767 (2)	0.66353 (17)	0.47953 (16)	0.0423 (5)	
H7	0.757186	0.685406	0.451533	0.051*	
C2	0.4871 (2)	0.51212 (16)	0.75445 (16)	0.0370 (5)	
C8	0.6844 (2)	0.61629 (17)	0.56270 (16)	0.0394 (5)	
H8	0.770224	0.606313	0.590012	0.047*	
C6	0.5498 (2)	0.67873 (16)	0.43719 (16)	0.0392 (5)	
C4	0.4384 (2)	0.59718 (17)	0.56390 (17)	0.0404 (5)	
H4	0.357922	0.574908	0.591632	0.048*	
C5	0.4319 (2)	0.64410 (18)	0.48042 (17)	0.0440 (5)	
H5	0.346335	0.652779	0.452346	0.053*	
C1	0.5487 (2)	0.4838 (2)	0.84654 (17)	0.0477 (6)	
H1A	0.559753	0.540211	0.884279	0.057*	
H1B	0.639951	0.457030	0.836228	0.057*	
C9	0.6958 (3)	0.7388 (2)	0.28174 (19)	0.0566 (7)	
H9A	0.691402	0.764360	0.220355	0.085*	
H9B	0.729678	0.674728	0.279361	0.085*	
H9C	0.757047	0.776878	0.318558	0.085*	

### Atomic displacement parameters (Å^2^)

**Table:**

	*U*^11^	*U*^22^	*U*^33^	*U*^12^	*U*^13^	*U*^23^
Cl1	0.0643 (5)	0.0892 (6)	0.0520 (4)	−0.0232 (4)	0.0053 (3)	0.0168 (4)
S1	0.0566 (4)	0.0723 (5)	0.0548 (4)	0.0139 (3)	−0.0006 (3)	0.0206 (4)
O1	0.0241 (7)	0.0781 (12)	0.0471 (10)	−0.0002 (7)	0.0034 (6)	0.0045 (9)
N1	0.0227 (8)	0.0513 (11)	0.0353 (10)	−0.0003 (7)	0.0005 (7)	−0.0016 (8)
C3	0.0295 (9)	0.0378 (11)	0.0327 (11)	0.0009 (8)	−0.0001 (8)	−0.0055 (9)
C7	0.0332 (11)	0.0514 (14)	0.0423 (13)	−0.0058 (9)	0.0034 (9)	0.0001 (11)
C2	0.0279 (10)	0.0441 (12)	0.0389 (12)	−0.0001 (8)	0.0028 (8)	−0.0050 (10)
C8	0.0274 (10)	0.0517 (14)	0.0391 (12)	−0.0021 (9)	−0.0020 (8)	−0.0020 (10)
C6	0.0400 (12)	0.0391 (12)	0.0385 (12)	0.0028 (9)	−0.0003 (9)	−0.0021 (10)
C4	0.0266 (10)	0.0506 (13)	0.0440 (13)	−0.0006 (9)	0.0008 (9)	−0.0011 (10)
C5	0.0299 (10)	0.0548 (14)	0.0475 (14)	0.0058 (9)	−0.0057 (9)	−0.0015 (11)
C1	0.0348 (11)	0.0638 (16)	0.0444 (14)	−0.0040 (10)	0.0040 (10)	0.0069 (12)
C9	0.0628 (16)	0.0592 (17)	0.0478 (15)	−0.0048 (12)	0.0032 (12)	0.0110 (12)

### Geometric parameters (Å, º)

**Table:**

Cl1—C1	1.757 (2)	C2—C1	1.515 (3)
S1—C6	1.764 (2)	C8—H8	0.9300
S1—C9	1.789 (3)	C6—C5	1.388 (3)
O1—C2	1.223 (2)	C4—H4	0.9300
N1—H1	0.82 (2)	C4—C5	1.379 (3)
N1—C3	1.414 (3)	C5—H5	0.9300
N1—C2	1.345 (3)	C1—H1A	0.9700
C3—C8	1.390 (3)	C1—H1B	0.9700
C3—C4	1.388 (3)	C9—H9A	0.9600
C7—H7	0.9300	C9—H9B	0.9600
C7—C8	1.378 (3)	C9—H9C	0.9600
C7—C6	1.388 (3)		
			
C6—S1—C9	103.42 (12)	C3—C4—H4	120.1
C3—N1—H1	116.1 (19)	C5—C4—C3	119.7 (2)
C2—N1—H1	115.1 (18)	C5—C4—H4	120.1
C2—N1—C3	128.60 (18)	C6—C5—H5	119.1
C8—C3—N1	116.83 (18)	C4—C5—C6	121.8 (2)
C4—C3—N1	124.31 (18)	C4—C5—H5	119.1
C4—C3—C8	118.9 (2)	Cl1—C1—H1A	108.9
C8—C7—H7	119.7	Cl1—C1—H1B	108.9
C8—C7—C6	120.6 (2)	C2—C1—Cl1	113.36 (16)
C6—C7—H7	119.7	C2—C1—H1A	108.9
O1—C2—N1	124.5 (2)	C2—C1—H1B	108.9
O1—C2—C1	122.62 (19)	H1A—C1—H1B	107.7
N1—C2—C1	112.76 (18)	S1—C9—H9A	109.5
C3—C8—H8	119.5	S1—C9—H9B	109.5
C7—C8—C3	120.9 (2)	S1—C9—H9C	109.5
C7—C8—H8	119.5	H9A—C9—H9B	109.5
C7—C6—S1	124.67 (18)	H9A—C9—H9C	109.5
C7—C6—C5	118.1 (2)	H9B—C9—H9C	109.5
C5—C6—S1	117.19 (17)		
			
S1—C6—C5—C4	−178.58 (19)	C2—N1—C3—C8	168.6 (2)
O1—C2—C1—Cl1	32.9 (3)	C2—N1—C3—C4	−11.7 (4)
N1—C3—C8—C7	−179.1 (2)	C8—C3—C4—C5	−0.8 (3)
N1—C3—C4—C5	179.4 (2)	C8—C7—C6—S1	178.92 (18)
N1—C2—C1—Cl1	−150.97 (18)	C8—C7—C6—C5	−0.7 (4)
C3—N1—C2—O1	8.9 (4)	C6—C7—C8—C3	−0.4 (4)
C3—N1—C2—C1	−167.2 (2)	C4—C3—C8—C7	1.2 (3)
C3—C4—C5—C6	−0.3 (4)	C9—S1—C6—C7	18.0 (2)
C7—C6—C5—C4	1.1 (4)	C9—S1—C6—C5	−162.3 (2)

### Hydrogen-bond geometry (Å, º)

**Table:**

*D*—H···*A*	*D*—H	H···*A*	*D*···*A*	*D*—H···*A*
N1—H1···O1^i^	0.82 (2)	2.06 (3)	2.875 (2)	174 (3)
C1—H1*B*···O1^i^	0.97	2.57	3.319 (3)	135
C4—H4···O1	0.93	2.32	2.903 (3)	121

Symmetry code: (i) *x*+1/2, *y*, −*z*+3/2.
